# BEACH-Gaze: Supporting Descriptive and Predictive Gaze Analytics in the Era of Artificial Intelligence and Advanced Data Science

**DOI:** 10.3390/jemr18060067

**Published:** 2025-11-12

**Authors:** Bo Fu, Kayla Chu, Angelo Ryan Soriano, Peter Gatsby, Nicolas Guardado Guardado, Ashley Jones, Matthew Halderman

**Affiliations:** Computer Engineering and Computer Science, California State University Long Beach, Long Beach, CA 90840, USA; kayla.chu@student.csulb.edu (K.C.); angeloryan.soriano@student.csulb.edu (A.R.S.); peter.gatsby01@student.csulb.edu (P.G.); nicolas.guardadoguardado@student.csulb.edu (N.G.G.); ashley.jones04@student.csulb.edu (A.J.); matthew.halderman@student.csulb.edu (M.H.)

**Keywords:** human computer interaction, eye tracking, gaze analytics, software and engineering

## Abstract

Recent breakthroughs in machine learning, artificial intelligence, and the emergence of large datasets have made the integration of eye tracking increasingly feasible not only in computing but also in many other disciplines to accelerate innovation and scientific discovery. These transformative changes often depend on intelligently analyzing and interpreting gaze data, which demand a substantial technical background. Overcoming these technical barriers has remained an obstacle to the broader adoption of eye tracking technologies in certain communities. In an effort to increase accessibility that potentially empowers a broader community of researchers and practitioners to leverage eye tracking, this paper presents an open-source software platform: *B*each *E*nvironment for the *A*nalyti*c*s of *H*uman *Gaze* (BEACH-Gaze), designed to offer comprehensive descriptive and predictive analytical support. Firstly, BEACH-Gaze provides sequential gaze analytics through window segmentation in its data processing and analysis pipeline, which can be used to achieve simulations of real-time gaze-based systems. Secondly, it integrates a range of established machine learning models, allowing researchers from diverse disciplines to generate gaze-enabled predictions without advanced technical expertise. The overall goal is to simplify technical details and to aid the broader community interested in eye tracking research and applications in data interpretation, and to leverage knowledge gained from eye gaze in the development of machine intelligence. As such, we further demonstrate three use cases that apply descriptive and predictive gaze analytics to support individuals with autism spectrum disorder during technology-assisted exercises, to dynamically tailor visual cues for an individual user via physiologically adaptive visualizations, and to predict pilots’ performance in flight maneuvers to enhance aviation safety.

## 1. Introduction

Eye tracking is increasingly being applied across multiple disciplines and sectors, such as diagnosing medical conditions [1], optimizing marketing and consumer research [2], enriching user experience with immersive interactions in virtual reality and augmented reality [3], enhancing automotive safety in advanced driver-assistance systems [4], and creating effective educational technologies enabled by gaze-driven learning behaviors [5]. Comprehensive overviews and guides in eye tracking research are extensively documented in [6], highlighting its diverse applications and methodological advancements. Coupled with recent advancements in machine learning, intelligent analysis and interpretation of gaze data will likely continue to drive innovation and discoveries in multiple domains that utilize eye tracking. However, the complexity involved in intelligent data analysis continues to be a barrier to the widespread adoption of eye tracking in certain communities, as such analyses typically require strong technical backgrounds. With eye tracking applications having grown to include the broader community of practitioners who may not always be readily equipped with comprehensive programming knowledge to develop tools independently, there is a pressing need for software support that can rapidly produce insights from gaze data. Increased accessibility to intelligent gaze analytics will thus facilitate broader participation in, and adoption of, eye tracking technologies across diverse disciplines.

To this end, contributing to providing comprehensive and easy-to-use tool capabilities that aid researchers across disciplines in intelligent gaze analytics without needing extensive technical skills, this paper presents an open-source software solution with integrated machine learning support to facilitate analyses of descriptive gaze measures and gaze-enabled predictions, namely, the Beach Environment for the Analytics of Human Gaze (BEACH-Gaze). The overall goal of BEACH-Gaze is to simplify the interpretation of gaze data to democratize access to this technology and to empower more researchers to leverage eye-tracking in their work. By lowering technical barriers and increasing accessibility, BEACH-Gaze will likely enable a wider range of researchers and practitioners to harness the power of intelligent gaze analytics, leading to accelerated innovative and impactful applications across various fields.

More specifically, BEACH-Gaze is a desktop application with a graphical user interface and is distributed under the GNU General Public License with a reuseable and extensible codebase. It consists of two main modules, including (i) the descriptive analytics module that provides sequential and summative descriptive gaze measures derived from raw eye gaze capturing gaze patterns of a moment in time as well as over time, and (ii) the predictive analytics module that provides a range of machine learning support for gaze-enabled classifications facilitating simulations of real-time predictions. With an overall goal to support collaborative development and rapid innovation in intelligent gaze analytics, the significance of this work lies in its contribution towards more accessible, comprehensive, and collaborative solutions to potentially accelerate research and discoveries in eye tracking research and applications across diverse disciplines. Notably, BEACH-Gaze enhances accessibility to intelligent gaze analytics and expands the scope and depth of gaze-enabled research. Additionally, the open-source software promotes collaborative development, allowing researchers to benefit from a shared pool of knowledge and tools with a reusable and extensible codebase, which can lead to more consistent and comparable research outcomes in future studies and applications.

Within the context of BEACH-Gaze, we distinguish between real-time and simulated real-time gaze modeling and predictions as follows. Real-time modelling refers to the continuous acquisition, processing, and interpretation of raw gaze data as it is being generated by the eye tracker during task execution. Simulated real-time modelling utilizes recorded gaze data in a time-sequenced manner to emulate the conditions of a real-time system. While the data is not generated live, the simulation preserves the temporal structure and progression of gaze data, allowing researchers to test prediction algorithms, evaluate system responsiveness, and refine thresholding strategies under controlled conditions. The key distinction lies in the temporal immediacy and system integration, where BEACH-Gaze focuses on providing a flexible environment for iterative testing and retrospective analysis. To this end, BEACH-Gaze supports simulated real-time modelling, enabling researchers to transition from offline experimentation to real-time deployment as system matures and application demands evolve.

This paper presents the design, architecture, and functional capabilities of BEACH-Gaze, including its comprehensive support for descriptive and predictive gaze analytics, integrated machine learning models, and a user interface to reduce technical barriers to empower a broader community of researchers to engage with eye-tracking research and application. In the current era of artificial intelligence and advanced data science, BEACH-Gaze aims to bridge the gap between complex computational methods and accessible, interdisciplinary research in eye tracking. The novelty of BEACH-Gaze lies in its ability to simulate real-time gaze-based predictions, support sequential and summative gaze analyses, and provide extensive classification and regression capabilities. These capabilities of BEACH-Gaze allow granular, time-sensitive insights and behavioral profiling, critical for applications in domains where understanding and responding to human attention and cognitive states in real time is essential. Moreover, its integrated machine learning models facilitate a wide range of predictive tasks. Unlike existing tools that often require extensive programming knowledge or offer limited analytical depth, BEACH-Gaze combines accessibility with advanced functionality, making it uniquely positioned to support interdisciplinary eye tracking research and application. To demonstrate its practical utility and cross-domain applicability, three distinct use cases in diverse fields (autism, physiologically adaptive visualization, and aviation safety) are also presented in this paper that serve to contextualize the relevance and effectiveness of BEACH-Gaze in supporting real-world interdisciplinary research and applications to accelerate scientific discovery, enhance human decision-making, and support user-centered innovation.

## 2. Related Work

Over the years, numerous commercial and open-source solutions have been developed to support eye tracking research and gaze data analysis. Due to concerns such as the cost of licensing renewals, limited customization options, dependency on vendors for updates and support, and the restrictions imposed by proprietary source code, there has been a growing need for open-source alternates within the scientific community. Some notable examples are discussed below.

In the context of facilitating open-source execution of eye tracking studies, PyGaze [7] offers a cross-platform toolbox designed for minimal-effort programming of eye tracking experiments written in Python. PyGaze aims to combine the ease of graphical experiment builders with the flexibility of programming, allowing researchers to create experiments with short, readable code without sacrificing functionality. It provides visual and auditory stimuli, collects data from a range of devices (e.g., keyboards, mice, joysticks), supports custom algorithms for eye movement detection, is compatible with multiple eye tracker brands (e.g., EyeLink, SMI, Tobii), and integrates Python libraries for enhanced functionality. Another example is GazeParser [8], an open-source and multiplatform library that provides low-cost eye tracking to more expensive commercial alternatives. It integrates and utilizes Python packages (e.g., OpenCV, SciPy, and Matplotlib) in data visualization, consists of a video-based eye tracker, and a set of Python libraries for data recording and analysis. GazeParser is compatible with experimental control libraries such as PsychoPy [9] and VisionEgg [10]. PsychoPy is an open-source software suite written in Python that is designed to create visual and auditory stimuli for neuroscience experiments. Though primarily developed for creating experiments in psychology, it also supports eye tracking experiments with extensive customization options for stimuli and experimental setups, is cross-platform compatible (e.g., Windows, macOS, Linux), supports simple scripts, and utilizes Python libraries such as OpenGL for graphics. VisionEgg is an open-source library for real-time visual stimulus generation. It is typically used in conjunction with eye tracking to create complex visual scenes by leveraging OpenGL. Written in Python with extensions in C, it supports real-time stimulus generation of complex visual scenes (e.g., 3D graphics), it provides luminance and temporal calibration, and accepts various input devices (e.g., mice, movement tracking systems, digital triggers). Originated from research in physics education, OGAMA [11] supports the recording and analyzing of eye tracking and mouse tracking data from slideshow-based experiments. It is written in C#.NET and is open source, with features supporting slideshow design, AOI definition, recording and replaying of eye and mouse tracking data. Recent efforts in developing open-source tools for running eye tracking experiments also include OpenGaze [12] that supports gaze and facial behavior analysis in web applications, Libretracker [13] that tracks eye movements using head-mounted webcams, and the TobiiGlassesPySuite [14] that supports the use of Tobii Pro Glasses 2 in platform-independent recordings.

To support evaluations and comparisons made across eye tracking systems and calibration methods, one example of an open-source and tracker-independent system is TraQuMe [15], which is designed to evaluate tracker reliability and data quality. It offers spatial accuracy and precision by measuring the proximity of the gaze point to the target and the consistency of repeated measurements. Additionally, it provides visualizations to help interpret results and works independently of specific eye tracker brands. Another example is GazeVisual-Lib [16], which is designed to evaluate the performance and data quality of eye trackers. It supports the extraction of evaluative metrics (e.g., true vs. estimated gaze position, de-noising and outlier removal) and provides visualizations (e.g., heatmaps, gaze plots, AOI specific metrics) for the researcher to thoroughly compare performance and identify limitations of their eye tracking systems.

In the context of open-source tools supporting gaze data analysis, one example is PyTrack [17], which offers support for both analysis and visualization of gaze data, such as automated extraction of gaze measures (e.g., blinks, fixations, saccades, microsaccades, and pupil size from raw gaze), visualizations (e.g., gaze plots, heat maps, and dynamic visualizations), statistical tests (e.g., *t*-tests and ANOVA tests), and area of interest (AOI) analysis (e.g., number of revisits in user-defined AOIs). To overcome challenges in eye tracking studies conducted on wide-field screens with natural head movements, ref. [18] presents an open-source toolkit with the Pupil Core eye tracker to support dynamic AOI analysis such as semi-automatic and manual allocation of dynamic AOIs, extractions of dwell times and time to first entry, as well as overlaying gaze data on video. EMDAT [19] is an open-source toolkit designed to support processing and analysis of raw data derived from Tobii eye trackers via Tobii Studio. It provides built-in data cleaning functions and can generate gaze features such as fixations, saccades, pupil metrics, and temporal patterns for each participant. It also supports batch processing of multiple participants. There are also several frameworks and toolboxes to support gaze data analysis including those written in MATLAB such as GlassesViewer [20] focusing on analyzing data from the Tobii Pro Glasses 2 eye tracker, SacLab [21] focusing on saccade analysis, EALab [22] focusing on multivariate data analysis and classifications, Eye-MMV [23] focusing on fixation detection using a novel two-step spatial dispersion threshold algorithm, GazeAlyze [24] focusing on static visual stimuli with extendable modules; others written in Python such as PSOVIS [25] that is designed to extract post-saccadic oscillations signals from the eye movement recordings and PyGazeAnalyzer [7] that extends PyGaze and supports high-level plotting of eye tracking data; as well as those written in R such as ETRAN-R [26] that supports visualization and statistical analysis of eye tracking data. Table 1 presents a comparison of the key features of these tools.

One key observation of the available open-source tools is that, despite their goal of reducing the technical effort for researchers, they often still necessitate some basic understanding of Python, MATLAB, or R. This requirement can pose a barrier for non-technical experimenters. To address this issue in an effort to broaden participation of eye tracking research and applications, BEACH-Gaze offers a graphical user interface (GUI) that eliminates the need for prior programming knowledge, thereby making it accessible to researchers and practitioners of all technical backgrounds. Another observation is that while there is substantial analytical support for extracting gaze activities, there is a lack of descriptive gaze measures that quantify and characterize sequential visual attention and gaze patterns. Current analytical support has predominantly emphasized summative gaze metrics that capture overall traits and tendencies across an entire recording. However, there remains a significant gap in analytical support for exploring the temporal dynamics of gaze data, thereby overlooking how the evolution of gaze over time can inform and enhance machine intelligence. To address this, BEACH-Gaze offers window-segmented sequential analyses of descriptive gaze measures, capturing the nature of time-series gaze data, reflecting the evolution of gaze with deeper insights into user behavior.

Another key observation is that, apart from EALab, there is a notable lack of classification support for generating gaze-empowered predictions to facilitate intelligent gaze analytics. Notably, BEACH-Gaze and EALab differ in several key aspects. Firstly, BEACH-Gaze enables temporal gaze analyses to quantify how gaze patterns evolve over time as a person’s visual needs change during an interaction. Secondly, recognizing the potential evolution of gaze in the same visual scene, BEACH-Gaze allows the experimenter to customize periodic analyses of descriptive gaze measures by setting timed, scheduled intervals, focusing solely on significant gaze events, or utilizing cumulative gaze tendencies exhibited by an individual. As gaze patterns evolve, the resulting classifications will also vary based on differences observed at different time intervals. As such, to support time-based predictions throughout an interaction, BEACH-Gaze allows the experimenter to customize the timing and frequency of classifications to simulate real-time gaze-driven predictions. Lastly, BEACH-Gaze supports a wider range of models (46 classification and 31 regression models compared to 6 classifiers in EALab) and does not require the experimenter to have expertise in configuring machine learning algorithms.

## 3. BEACH-Gaze

BEACH-Gaze is compatible with Windows and MacOS and supports the processing and analyses of raw gaze data generated from the Gazepoint GP3 and GP3 HD eye trackers [27]. By default, it is configured to process raw gaze data collected from monitors measuring 24″ with full HD resolution 1920 × 1080 pixels, which are the highest specifications supported by the Gazepoint eye trackers. BEACH-Gaze is written in JAVA and freely available to download at [28]. BEACH-Gaze supports individual (i.e., one person’s gaze) and batched (i.e., a group of individuals’ gazes) processing and analyses of raw gaze, analysis of experimenter-defined AOIs, descriptive gaze measures at timed intervals and classifications thereof to simulate real-time gaze-enabled predictions.

### 3.1. Design Overview

**Figure 1:**
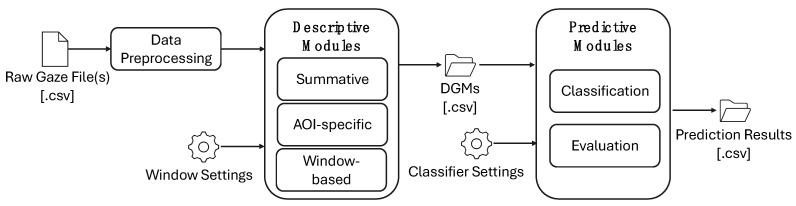
Architecture of BEACH-Gaze.

Figure 1 illustrates the architecture of BEACH-Gaze. Raw gaze files (e.g., all_gaze.csv and fixations.csv) first undergo a pre-processing process to reduce noise, whereby invalid entries (as indicated by the eye tracker’s validity codes, negative values, and off-screen entries), incomplete entries (e.g., when only one eye was captured or when x and y coordinates are missing), and corrupted entries (e.g., pupil dilation exceeding possible ranges) are removed. Anisocoria (asymmetric pupils) is rarely greater than 1 mm [29], and normal pupil size in adults typically varies from 2–4 mm in diameter in bright light and 4–8 mm in the dark [30].

Once de-noised, the data is further processed by the Descriptive Modules to produce descriptive gaze measures (DGMs, discussed in Section 3.2). Depending on the researcher’s configuration, one, two, or all modules may be executed. In the summative module, cumulative DGMs are generated for the entire duration of an eye tracking recording. This can be used to support researchers interested in the aggregated gaze behavior over time that provides an overview of visual attention patterns by consolidating spatial and temporal gaze data into a single, comprehensive view. For researchers interested in the temporal progression of gaze, such as nuanced shifts in attention that may have occurred throughout an interaction, the window-based module supports the generation of temporal DGMs at various time intervals, capturing the evolution of gaze throughout an interaction. If the researcher had defined AOIs (reflected in the raw gaze data), then DGMs can also be generated for the specific AOIs, in either summative fashion (i.e., aggregated results of an entire recording) or temporal fashion (i.e., sequentially segmented results throughout a recording).

To support intelligent analytics of gaze, summative, window-based, and AOI-specific DGMs can be sent to the *Predictive Modules* to generate predictions for a variable that is meaningful in the given experimental scenario, such as whether a participant’s task score will be above or below a threshold, or how long a participant will fixate on a visual cue based on the DGMs produced from the Descriptive Modules. The researcher can also configure whether classification or regression is to be performed, and the predictions, along with their accuracies, are produced. Further details on the modules, DGMs, window-segmentation of temporal gaze analytics, as well as the classifications are discussed below.

### 3.2. Descriptive Gaze Analytics

Table 2 presents the list of DGMs generated by BEACH-Gaze based on raw gaze with an overall goal of providing a comprehensive overview of the key gaze characteristics found in the raw gaze dataset. These DGMs can be generated for each researcher-defined AOI. Additionally, BEACH-Gaze can process raw gaze files on a per-person basis producing DGMs for that specific individual, as well as in batches on a group basis to produce aggregated DGMs for an entire group of individuals. The AOI-specific DGMs are inspired by the extensive review on various gaze measures documented in [31].

For a person or a group of people, the DGMs aim to summarize the different quantifiable aspects of the eye tracking dataset. For instance, where applicable, a set of descriptive statistics, e.g., sum, mean, median, standard deviation (SD), minimum, and maximum values, are calculated to help the researcher to understand the distribution, central tendency, and variability of a gaze dataset. In the context of saccades, such descriptive statistics can be applied to magnitude, duration, amplitude, velocity and peak velocity, as well as relative and absolute directions of valid data points captured over time. To determine peak velocity, BEACH-Gaze implements the algorithm proposed by [32] and approximates saccade amplitude via the Euclidean distance between fixations [33,34]. Similarly, a set of DGMs are determined for fixations that describe the fundamental characteristics of the gaze dataset such as its spread, central, and range. In addition, the smallest boundary that can wrap around all fixations is calculated via convex hull to indicate the area within which a person’s (or a group of people’s) gaze has moved.

To support gaze analytics for eye tracking studies involving AOIs, BEACH-Gaze produces a range of AOI-specific DGMs. To quantify how a person (or a group of people) allocates visual attention across various AOIs, BEACH-Gaze generates stationary entropy to indicate how evenly gaze is distributed across different AOIs, and transition entropy to measure the randomness of transitions between different AOIs as defined by [35] and implemented in [36]. In addition, the proportion of fixations and their durations spent in an AOI relative to all fixations captured is generated. To quantify how a person (or group of people) navigates across various AOIs, BEACH-Gaze generates statistics such as count and proportion of gaze travelling from one to the other AOI in each pairwise AOI. Note that for a pair of AOIs named A and B, transitions from A to B differ from transitions from B to A. Also, when determining proportions, self-transitions are defined as gaze moving away from an AOI and returns to the same AOI without visiting any other AOIs in between. Moreover, to capture sequences of AOIs, as well as emerging subsequence patterns in a person’s (or a group of people’s) gaze, BEACH-Gaze implements the algorithms proposed by [37]. A sequence is defined as the ordered series of AOIs that make up a person’s entire scanpath, and BEACH-Gaze uses the algorithm proposed in [38] to extract subsequence patterns from these sequences, in both expanded and collapsed forms. An expanded pattern includes all fixations, including consecutive fixations within the same AOI (i.e., repetitions), whereas collapsed patterns discard AOI repetitions. For example, with five AOIs named A, B, C, D, and E, the sequence ACCDEABAAAABC becomes ACDEABABC in its collapsed form and remains changed in its expanded form. Additional measures such as how often specific patterns occur within the AOI sequences of a group of people (i.e., pattern frequency), how frequent a particular pattern of AOIs appears across a group of people (i.e., sequence support), the mean occurrences of a specific pattern across all sequences analyzed (i.e., mean pattern frequency), and the relative frequency of a specific pattern within the entire set of gaze sequences (i.e., proportional pattern frequency) can then be determined using BEACH-Gaze.

Lastly, BEACH-Gaze supports the generation of DMGs such as blinks per minute (i.e., blink rate), dynamic change in pupil size (of each eye and across both eyes), the relationship between the time spent on processing information and the time spent on information search (i.e., fixation-to-saccade ratio), and the total time of eye movements including both fixations and saccades (i.e., scanpath duration). To determine pupil dilation, a baseline can be set by the researcher via the GUI (discussed in Section 3.4), where a person’s pupil sizes are observed over a period of time during relatively low-demand task conditions (e.g., during calibration). Subsequent enlargement of the pupils can then be determined compared to the baseline values.

### 3.3. Evolution of DGMs Captured via Window Segmentation

**Figure 2:**
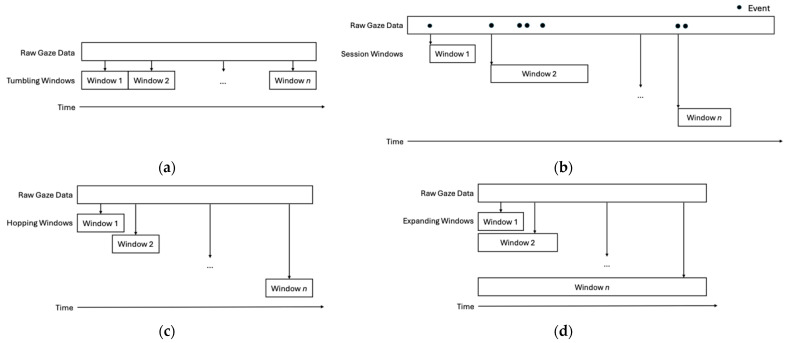
Window-based DGM Analytics to Facilitate Real-Time Classifications: (**a**) Scheduled Digests via Tumbling Window; (**b**) Irregular Events via Session Window; (**c**) Gaze Snapshots via Hopping Window; (**d**) Cumulative Gaze via Expanding Window.

BEACH-Gaze allows the experimenter to tailor periodic analytics of DGMs, supporting temporal gaze analyses aimed at reflecting gaze evolution over time as a person’s visual needs evolve throughout an interaction, which ultimately facilitates simulations of real-time gaze-based predictions. To achieve this, BEACH-Gaze provides four window-based methods to segment and configure periodic generations of DGMs (Figure 2), namely scheduled digests via tumbling windows, cumulated gaze via expanding windows, current snapshot via hopping windows, and irregular gaze events via session windows. In all windows, the researcher can customize the window size and timed interval as appropriate depending on the needs and goals of a given scenario.

To capture gaze as scheduled digests, BEACH-Gaze supports DGM analytics performed in a series of non-overlapping and fixed-in-size tumbling windows at scheduled contiguous time intervals (Figure 2a). For example, if the size of the tumbling window is set to 30 s, then the first window would contain DGMs for gaze collected between 00:00:00 and 00:00:30, the second window would contain DGMs for gaze generated between 00:00:30 and 00:00:60, and so on until the end of all known gaze have been analyzed.

Alternatively, the researcher can emphasize irregular gaze events detected for a person (e.g., elevated values compared to an established baseline) that can be analyzed in a series of non-overlapping and non-fixed-in-size session windows (Figure 2b) with a specific timeout duration and a maximum size. In this context, irregular gaze events are significant deviations in DGMs from a baseline specific to an individual or task. For instance, during calibration or knowing an established norm for a given task, a baseline presenting typical DGM values can be generated for a person or a specific task. Subsequent DMGs can then be compared to this baseline, whereby increases in value would be considered as irregular gaze events. For example, by observing a person during the initial phase of a task, one can establish a baseline profile of gaze behavior characterized by typical patterns and tendencies, e.g., average fixation duration, average blink rate, etc. Subsequent values are then evaluated relative to this baseline profile, where increases may be interpreted as notable deviations (i.e., events). The determination of what constitutes a meaningful deviation can be determined by the researcher and in consideration of the task nature. As an example, with a session window set to a two-minute timeout and a maximum five-minute duration, the first window is created after detecting the initial irregular gaze event and continues to search for the next event for two minutes. If no further events are found, the window ends after two minutes. If another event is found, the window renews search for two more minutes, either times out when no further events are found or ends once reaching the maximum duration of five minutes.

Moreover, the most recent gaze state of a person can be reflected in their gaze snapshots, where the last known gaze is analyzed via overlapping and fixed-in-size hopping windows (Figure 2c) configured with a window size and hop size. For example, with a 90 s window size and 60 s hop size, the first window would contain DGMs for gaze collected during 00:00:00 and 00:01:30, the second window would contain DGMs between 00:01:00 and 00:02:30, and so on until reaching the end of all known gaze.

Lastly, BEACH-Gaze supports cumulated analytics that can be processed via overlapping and non-fixed-in-size expanding windows (Figure 2d). For example, with a window size configured to be three minutes, the first window would contain DGMs for gaze collected during 00:00:00 and 00:03:00, the second window would then expand to contain DGMs captured between 00:00:00 and 00:06:00, so on, and the last window would contain DGMs for the entire gaze dataset generated during an interaction.

### 3.4. Predictive Gaze Analytics

In addition to providing descriptive gaze measures, BEACH-Gaze builds upon Waikato Environment for Knowledge Analysis (WEKA) [39] and integrates a range of established classification models in machine learning to support advanced gaze-enabled predictions. With window-based DGMs that can be generated throughout various stages of an interaction, the resulting classifications will also vary depending on the differences observed at various time intervals. The researcher can input DGMs into the Predictive Gaze Analytics of BEACH-Gaze to generate classifications to predict a discreet category (e.g., if a person belongs to the successful or unsuccessful group) or a continuous value (e.g., a task score) based the DGMs captured using one or more windows. To support these gaze-driven predictions, BEACH-Gaze integrates the WEKA API version 3.8.6 [40] and leverages a broad range of established classification models (outlined in Table 3) to generate classifications, with default WEKA configurations in the classification models (that can be customized) using a stratified 10-fold cross validation for model evaluation and Bonferroni-corrected *t*-tests for statistical testing to ensure robust and reliable performance.

### 3.5. User Interface

**Figure 3:**
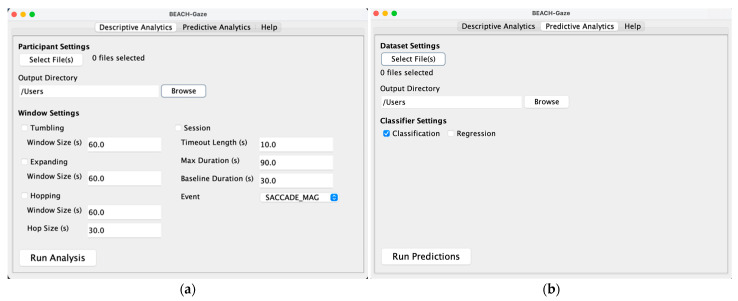
Graphical User Interface in BEACH-Gaze: (**a**) Descriptive Gaze Analytics; (**b**) Predictive Gaze Analytics.

Figure 3 shows the GUI in BEACH-Gaze, highlighting the two main modules that generate descriptive (Figure 3a) and predictive analytics (Figure 3b). In the Descriptive Analytics tab, the researcher would first input the raw gaze files (either for one person or batched for a group of people) and select the desired output directory for the DGMs, then proceed to choose one or more of the windows to run analysis. As discussed in Section 3.3, timed and scheduled DGMs would be generated in a temporal fashion to capture sequential gaze measures detected at various stages. If none of the windows is selected, one summative file containing all DGMs for the entire duration is generated (instead of sequential DGM files at various time intervals). The researcher can then perform classification experiments in the Predictive Analytics tab, by providing the sequential DGM files (e.g., the output from Descriptive Analytics) and setting the type of classification models to apply (e.g., classification or regression) depending on the goal of the prediction.

In the case of a tumbling window (as illustrated in Figure 2a), the DGMs and consequently the predictions based upon them are generated using gaze collected in a researcher-defined time zone (e.g., a window size of 60 s is set in an example shown in Figure 3a, which contains gaze data captured during the initial 0–60 s); it then moves onto the next bordering time zone to generate subsequent DGMs and predictions (i.e., based on gaze data captured between 60–120 s, then 120–180 s, and so on), and tumbles forward until reaching the end of an interaction.

In the expanding window (as illustrated in Figure 2d), an initial set of gaze data is analyzed (e.g., an example shows 60 s in Figure 3a, meaning the first window contains gaze data captured during the initial 0–60 s), which then gets expanded to include new gaze data at the next specified time interval (i.e., 0–120 s, followed by 0–180 s, and so on).

In the case of a hopping window (as illustrated in Figure 2c), BEACH-Gaze processes gaze using a researcher-defined window size, then moves forward to the next scheduled hop relative to the previous one. The example shown in Figure 3a has a 60 s window size and a 30 s hop size, meaning every 30 s, gaze over the last 60 s is analyzed (i.e., DGMs and predictions based upon are generated using gaze captured between 0–60 s, 30–90 s, 60–120 s, and so on).

Lastly, throughout an interaction, a person may encounter pivotal moments that significantly influence their performance. These moments can manifest as distinct gaze behaviors, reflecting phases of irregular gaze events. In this context, gaze events are essentially deviations (i.e., subsequent values exceeding a baseline) from the established norms (i.e., a baseline that can be determined during calibration, or at the start of an interaction) of a person’s gaze. BEACH-Gaze supports a number of approaches to detect an irregular gaze event, including saccade magnitude, saccade direction, left eye pupil diameter, right eye pupil diameter, average of left and right eye pupil diameter combined, fixation duration, and blink rate. When using the session window (as illustrated in Figure 2b), the analytics begin when the first event is found, and it then keeps searching for the next event within a specified time period. An example in Figure 3a shows a 10 s timeout, 90 s maximum duration, 30 s baseline duration, and the SACCADE_MAG in the dropdown menu, meaning that using the saccade amplitude to detect irregular gaze events, a baseline (i.e., the average saccade amplitude observed) is established after 30 s, whereby subsequent saccade amplitudes that are higher in value are deemed as “events”. When another event is found, the session window grows (i.e., the 10 s timeout is renewed) until it meets the maximum duration set to 90 s. If no further events are found, the session window closes.

## 4. Use Cases

BEACH-Gaze has contributed to technology innovation and knowledge discovery across several application areas that leverage eye tracking. Three example use cases are discussed below, illustrating example applications of descriptive and predictive gaze analytics, including (i) supporting technology-assisted exercise applications aimed at increasing physical activity intensity for individuals with autism spectrum disorder (discussed in Section 4.1); (ii) enabling physiologically adaptive visualizations that dynamically respond to an individual’s gaze (discussed in Section 4.2); and (iii) enhancing aviation safety by predicting pilot performance during flight maneuvers based on DGMs, thereby informing the timing of critical interventions (discussed in Section 4.3). These case studies aim to highlight the critical role of gaze analytics in enhancing machine intelligence—enabling systems to interpret human attention, adapt in real time, and make informed predictions—thereby advancing the synergy between human users and machine empowered intelligent decision-making in the era of artificial intelligence and advanced data science.

### 4.1. Technology Assisted Exercise for Individuals with Autism Spectrum Disorder

The integration of exercise and technology has sparked the emergence of digital fitness since the early 2000s, with innovations designed to inspire greater physical activity [41]. Users of fitness devices and applications have often found these technologies effective in encouraging and sustaining physical activity, leading to enhanced health and overall well-being. However, a significant number of tools often fall short for certain groups, such as individuals with autism spectrum disorder (ASD). Research in the U.S. found that 1 in 54 children were diagnosed with ASD as of 2016 [42], characterized by deficits in social communication, restrictive interests, and repetitive behaviors [43]. Consequently, individuals with ASD are more likely to lead sedentary lifestyles, increasing the risk of obesity and other health issues [44].

Given these factors, there is a pressing need to promote equitable access to sport by advancing technology assisted exercise apps that encourage physical activity amongst individuals with ASD. Technology-assisted exercise applications, such as those described in [45,46], leverage real-time heart rate visualizations to encourage ASD individuals to participate in longer and more intense physical activities, both in single-user and multi-user modes. More specifically, while wearing a Scosche heart rate monitor that measures heart rates in beats per minute (BPM), a user would begin an exercise session with as a main character flying forward a path, as the heart rate elevates or drops in real time. The goal is to enrich the user experience of a physical exercise, through technology, to increase motivation and promote engagement for individuals with ASD.

To evaluate the effectiveness of such an application, the researchers conducted a series of eye tracking usability studies involving 20 verbal individuals with ASD (all of whom were able to read and comprehend the instructions provided). Each participant completed two stationary bicycle exercise sessions (on a Matrix IC7 Indoor Stationary Bicycle) on separate days: one control session without the application that visualizes heart rates in real time, and one experimental session with the application. The order of these sessions was randomized and counterbalanced across participants to mitigate order effects.

**Figure 4:**
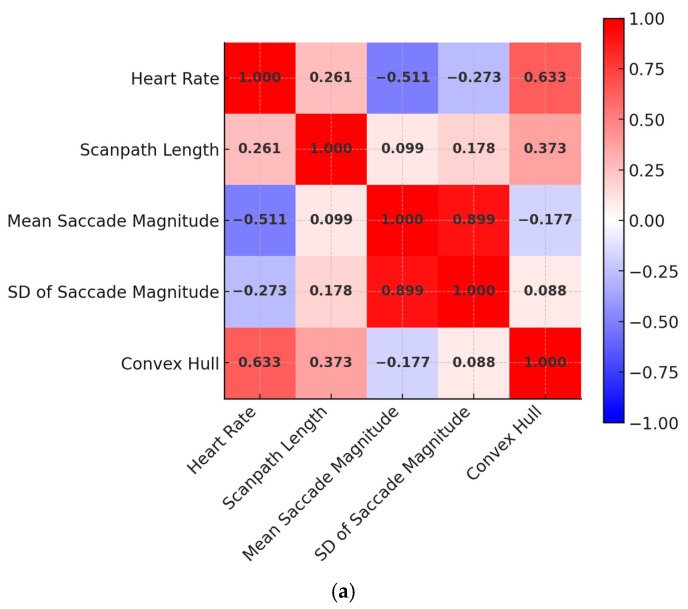

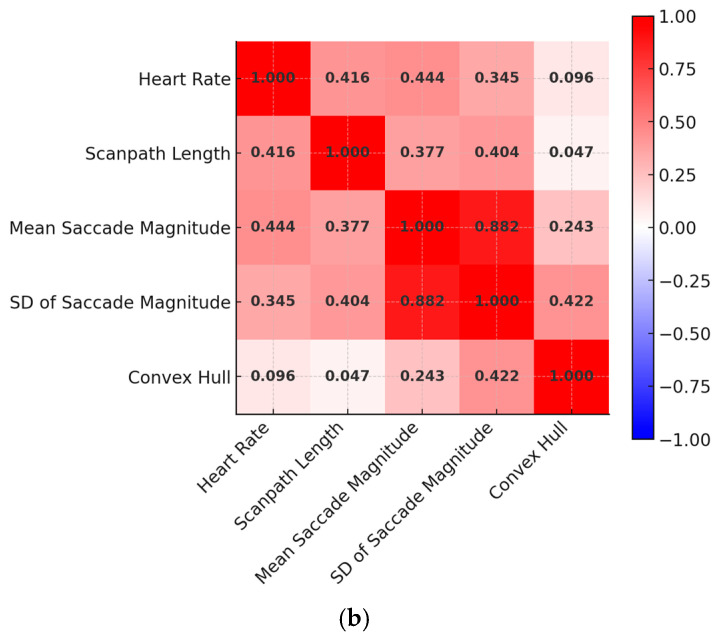
Heat map matrices showing correlation coefficient (r values) generated between pairwise DGMs and heart rates in evaluative studies using eye tracking: (**a**) r values generated for the above median group; (**b**) r values generated for the below median group.

The results indicated that amongst individuals with ASD, 83% achieved higher heart rates, 66.6% maintained heart rates at or above 90 BPM, and 27.7% re-engage in their exercise to reach 90 BPM after previously dropping below. To further investigate how the individuals interacted with the given iPadOS application, the participants were grouped into two categories using a median split of their heart rates achieved during the exercise (at 118 BPM), as the above median group and the below median group. DGMs produced by BEACH-Gaze showed several key differences in the visual attention between the two groups, as shown in Figure 4. For instance, those individuals who achieved higher heart rates searched for visual cues that were relatively close to one another (as indicated by the Pearson correlation coefficient r value of −0.511 between heart rate and mean saccade magnitude), suggesting a more consistent and controlled interaction. This is further amplified by the SD of the saccade magnitudes (with an r value of −0.273), indicating that those who achieved higher heart rates exhibited less dispersed searches, suggesting more focused gaze behaviors. Furthermore, individuals with above the median heart rate generated longer scanpaths (with an r value of 0.261), suggesting an increased engagement with the iPadOS application. Similarly, positive correlations between heart rates and convex hull areas were found (with an r value of 0.633), indicating the individuals achieved higher heart rates also scanned a larger area as they interacted with the iPadOS application. Notably, while negative correlations are evident for the above median group (as heart rate increased, both mean saccade magnitude and SD of saccade magnitude decreased, shown in Figure 4a), only positive correlations are found for the below median group (as heart rate increased, both mean saccade magnitude and SD of saccade magnitude increased, shown in Figure 4b). This finding suggests that individuals who were less successful in the exercise may have shown lower engagement with the iPadOS application, indicated by their dispersed DGMs. This, in turn, highlights the potential benefits of the proposed technology-assisted application in its effectiveness to engage individuals with ASD, thereby enhancing exercise intensity and supporting the achievement of physical activity goals.

### 4.2. Physiologically Adaptive Visualization for Mappings Between Ontologies

**Figure 5:**
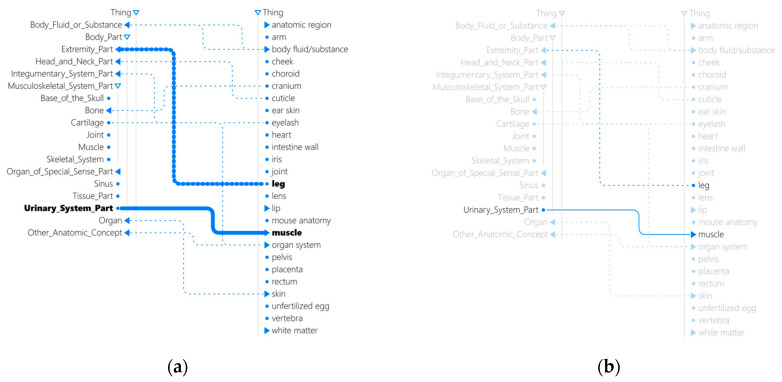
Adaptive Visualization Driven by Real-Time Gaze-Based Predictions of User Failure: (**a**) An example of highlighting upon predicted user failure; (**b**) An example of deemphasis upon predicted user failure. Clicking on a node toggles the expansion or collapse of an ontological class. Solid triangles represent nodes with children, hollow triangles indicate nodes that are fully expanded in the visualization, and dotted nodes signify classes without children. Solid lines between nodes denote mappings between classes that are fully visible in the visualization, e.g., “Urinary_System_Part” in one ontology is mapped to “muscle” in another ontology in (**a**). Dotted lines represent mappings between subclasses where at least one class is not currently visible in the visualization, e.g., “leg” is mapped to a subclass of “Extremity_Part” in (**b**).

Traditional visual aids to support human interaction with structured datasets such as ontologies have typically adopted one-size-fits-all solutions, overlooking personalized visual cues to enhance human comprehension of complex data and ontological relationships. Contributing to advancing adaptive visualizations for mappings between pairwise ontologies, a physiologically adaptive visualization that customizes visual cues for an individual user based on this person’s eye gaze is shown in Figure 5. The goal of an adaptive visualization system such as that in [47,48] is to leverage signals in eye gaze to predict a user’s success in a given task. If a potential failure is predicted, real-time visual interventions by means of highlighting key elements or de-emphasizing distractions are triggered to guide the user’s attention and support task completion.

Contributing to recognizing *when* timely interventions should be invoked, a series of experiments utilizing BEACH-Gaze has demonstrated [49,50,51,52,53,54] the benefits of comparing different approaches to window segmentation in sequential gaze analytics when generating user predictions in the domain of human-semantic data interaction. Building upon the knowledge gained across different classification models and influential gaze measures that predict *when* adaptations should be initiated, the gaze-adaptive visualization [47,48] advances personalized visualization to provide solutions that also recognize *what* (e.g., adapting to an individual’s performance) and *how* (e.g., displaying visual overlays dynamically in real time) to adapt to an individual user. More specifically, adaptive visualization is achieved using a long short-term memory network to continuously predict a user’s task success and failure based on real-time gaze collected while a person is interacting with the visualizations. When a task failure is predicted, visual interventions (e.g., highlighting and deemphasis) are applied to direct user attention and aid task completion. Empirical evaluation of this adaptive visualization with 76 participants in a between-subject study has indicated improved user performance without tradeoffs in workload or task speed.

### 4.3. Enhanced Aviation Safety via Gaze-Driven Predictions of Pilot Performance

Commercial air travel is widely regarded as one of the safest modes of transportation today, with fewer than one accident per million departures [55]. Over the past few decades, the number of aviation accidents has steadily declined due to technological advancements such as automation, along with enhanced training and improved air traffic control procedures [56]. However, over-reliance and overconfidence in automated systems has potentially resulted in a lack of manual and active monitoring by flight crews, with 60–80% of aviation accidents attributed to human error [57]. Effective and efficient monitoring is vital for aviation safety, particularly during dynamic phases such as takeoff and landing, whereby accurately observing various flight instruments and integrating multiple sources of readings and visual cues are crucial for decision-making. Since most of this visual information is processed by the human eyes, there is an opportunity to investigate the feasibility of incorporating eye tracking into human-centered flight deck designs.

A first step towards realizing future intelligent aircraft that can potentially anticipate and mitigate threats at runtime is to identify the optimal timing for system intervention such as if a pilot will succeed or fail while performing a flight maneuver. To this end, BEACH-Gaze has enabled predictive analytics of pilots’ gaze in simulated flight scenarios. In a study involving 17 participants asked to take off in a Cessna 172 aircraft equipped with the six-pack instrument panel on the X-Plane 11 simulator, results showed that it was feasible to predict pilots’ performance in the takeoff with up to 83.5% accuracy across a range of established classifiers [58]. Also, the DGMs found to be most influential to predict a pilot’s performance in the takeoff included less dispersed gaze magnitudes, longer average saccadic magnitude, longer scanpaths, and larger convex hulls. Furthermore, pilots who performed well during the climb phase demonstrated quicker visual searches, those who performed better during the takeoff phase exhibited a wider scanned area of their visual environment, and more successful pilots reported lower cognitive workload that is also reflected in their pupil dilations [59].

**Figure 6:**
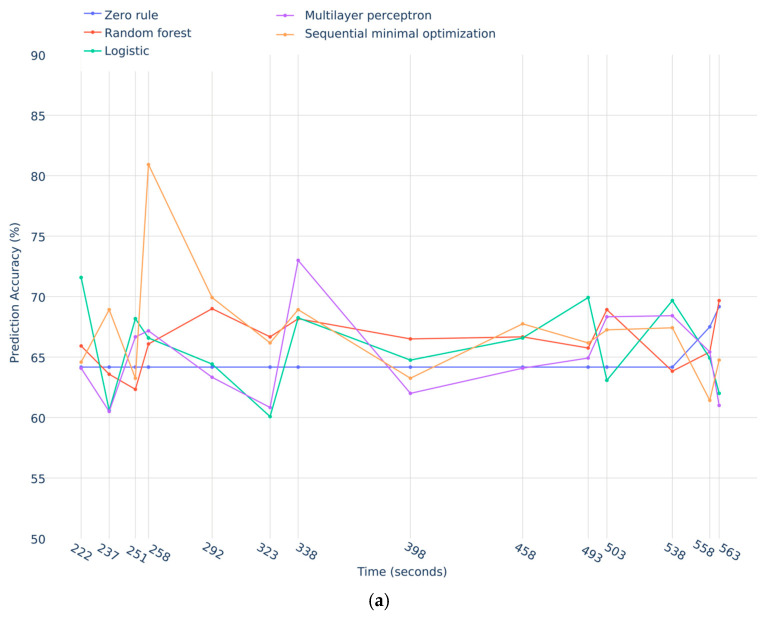

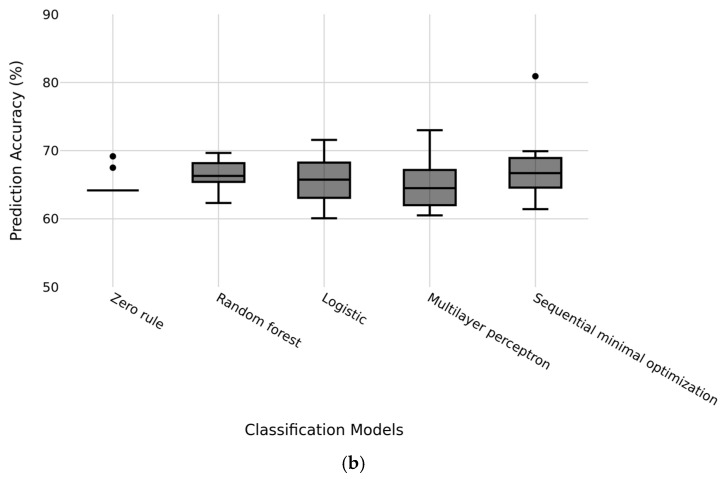
Predicting Pilot Success and Failure in Simulated Approach and Landing over Time using Average Fixation Duration as the Baseline to Detect Irregular Gaze Events: (**a**) Prediction accuracies of multiple classifiers; (**b**) Performance distribution of the classification models.

In another X-Plane 12 simulated study [60] involving 50 pilots performing an Instrument Landing System (ILS) approach in cloudy conditions landing a Cessna 172 aircraft at the Seattle-Tacoma International airport, BEACH-Gaze enabled the comparison of seven different approaches to detect notable gaze events experienced by a pilot, such as elevated values of selected DGMs including saccade magnitude, saccade direction, pupil dilation, fixation duration, blink rate, stationary and transition entropy. The results showed the effectiveness of leveraging upon session windows to detect notable gaze collected at pivotal moments of a given task when predicting pilot performance. As shown in Figure 6a, pilot success and failure can be predicted as early as 3.7 min after the task began, with accuracies up to 80.92% (after 4.3 min) using fixation duration to detect notable gaze events experienced by the pilots. Several established classifiers without special configurations outperformed a baseline classifier that predicts the majority (e.g., zero rule), with the support vector machine classifier (e.g., sequential minimal optimization) producing predictions with higher accuracies.

**Figure 7:**
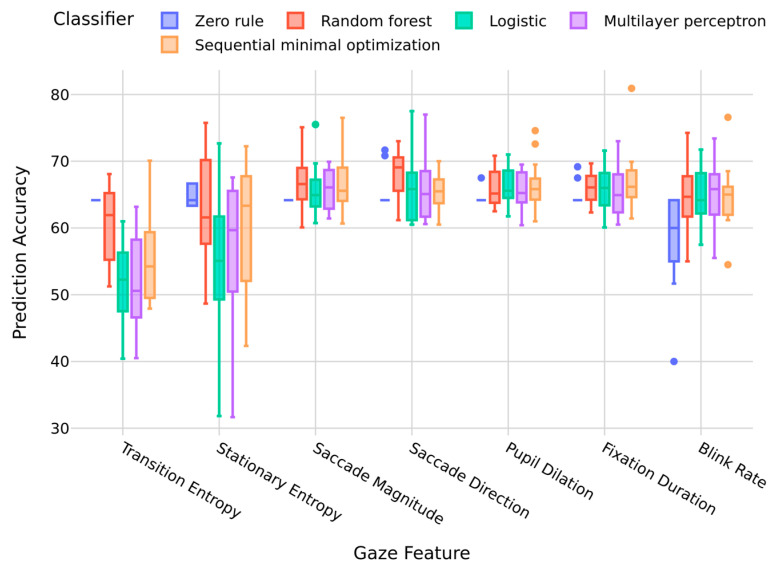
Classifier Performance Across Gaze Features when Predicting Pilot Success and Failure in the ILS Approach using a Session Window.

Demonstrating that capturing notable gaze behaviors can reflect key phases of critical events potentially indicating a pilot’s overall performance in the ILS approach, a session-window-based approach to predict task success and failure (binary categorical outcome either above or below a performance threshold) was evaluated in [60]. Specifically, gaze features available at runtime, such as saccade magnitude and direction, pupil dilation, fixation duration, and blink rate were used to detect irregular gaze events. A baseline (average values generated for these gaze features) for a pilot was established after observing the person for the first two minutes after task initiation. A session window was empirically determined and mapped to a four-second timeout and a sixty-second maximum duration to detect notable events. The rationale is that at the start of a task, cognitive demand is minimal, allowing for the capture of baseline behaviors reflecting a pilot’s typical procedures in visual search and information processing. As the task becomes increasingly demanding and complex (leading to increased mental stress and workload), increases from the baseline values (i.e., defined as notable events) may highlight critical moments during flight maneuvers that can be leveraged to predict the pilot’s performance. The predictions showed improved accuracies across a range of classifiers when compared against results derived from more established gaze metrics such as stationary and transition entropy (shown in Figure 7).

Compared to the zero rule classifier as a benchmark that predicts the majority class, consistently yielding an accuracy of approximately 64% with minimal variance across all features, all other classifiers showed varied levels of improvement in their prediction accuracies. Random forest demonstrated superior performance across most gaze features, particularly in stationary entropy, saccade magnitude, and blink rate. It achieved high median accuracy with relatively narrow interquartile ranges, indicating both effectiveness and stability. Sequential minimal optimization also performed robustly, especially when combined with fixation duration and pupil dilation. Logistic regression and multilayer perceptron exhibited feature-dependent performance, where the former exceled when combined with blink rate and pupil dilation, and the latter showed strong results when combined with saccade magnitude and fixation duration. Gaze features including saccade magnitude, pupil dilation, and fixation duration emerged as the most discriminative, enabling higher prediction accuracies across classifiers. Overall, all gaze features led to improved prediction accuracies across classifiers when compared to established gaze measures such as transition and stationary entropy.

## 5. Future Work

This paper presents BEACH-Gaze that is designed to simplify the technical aspects of gaze data analysis, therefore making eye tracking more accessible to a broader scientific community. In an era marked by rapid advances in artificial intelligence, machine learning, and the growth of large datasets, this work seeks to lower technical barriers, enabling broader use and participation of eye tracking technology. It also promotes accessible, intelligent analysis of gaze data across disciplines, supporting the development of machine intelligence and gaze-enabled intelligent systems.

BEACH-Gaze can be extended significantly in future development to provide robust support aiding the broader community interested in eye tracking research and applications. Several development work items are in the pipeline to improve and extend its features and functionalities. In particular, we plan to incorporate advanced deep learning models in BEACH-Gaze to enhance the precision and reliability of gaze-based predictions and classifications. This will likely enable more sophisticated analysis and interpretation of eye tracking data, opening up new possibilities in gaze-enabled intelligent systems, particularly in complex and dynamic interaction scenarios where traditional models may fall short. Also, we plan to support multi-task learning in more advanced predictive analytics, whereby BEACH-Gaze can perform multiple related tasks simultaneously (e.g., classifying gaze behavior while also predicting user intent), enriching the analytical depth and improving system efficiency.

Moreover, we intend to include visualizations to support graphical data analysis, such as static and dynamic heatmaps, gaze plots, time-series graphs, and group comparisons. These visualizations will enable more intuitive and powerful ways for researchers and practitioners to visually explore, analyze, and interpret gaze data, making it easier to gain actionable insights and identify patterns, trends, and insights otherwise hidden with traditional analysis methods. Furthermore, it is possible to explore the semantic interpretation of gaze as a communicative modality. Beyond its role in reflecting cognitive processes, eye gaze may also be interpreted as a sophisticated non-verbal language that could be used to construct a dictionary of gaze-based expressions [61], whereby DGMs could be mapped to communicative intent. As such, it may be possible to integrate semantic modeling to uncover patterns in gaze behavior that correspond to meaningful communicative signals to further enrich the interpretation of gaze semantics and deepen our understanding of human intent, particularly in social, educational, and clinical contexts.

Lastly, we aim to expand compatibility to support a wider range of eye trackers to make BEACH-Gaze more accessible to a broader audience. This includes integration with well-known eye tracking hardware from notable manufacturers to accommodate the diverse needs and preferences of researchers and practitioners in the field. The overall goal of these enhancements is to collectively make BEACH-Gaze a more powerful and versatile tool to facilitate a deeper understanding of gaze data and to foster innovation in gaze analytics across multiple domains, research scenarios, and user needs. Additionally, while this paper focuses on the design, architecture, functional capabilities, and cross-domain applicability, future evaluation focusing on empirical usability studies of distinct user groups (e.g., technical vs. non-technical users) can further quantify the effectiveness, efficiency, and user satisfaction of BEACH-Gaze.

## Figures and Tables

**Table 1:** Feature Comparison of Open-Source Tools for Gaze Data Analytics.

	GUI	Temporal Segmentation	Machine Learning	Compatible Eye Trackers	License
PyTrack	Data visualizations only	-	-	EyeLink, SMI, Tobii	GPL-3.0
[17]	-	-	-	Pupil Core	GPL-3.0
EMDAT	-	-	-	Tobii	BSD-2
Glasses Viewer	✓	-	-	Tobii Pro Glasses	CC BY 4.0
SacLab	✓	-	-	ViewPoint	BSD
EALab	✓	-	✓	Tobii, SMI	BSD
Eye-MMV	Data visualizations only	-	-	ViewPoint	GPL-3.0
GazeAlyze	✓	-	-	Not specified	GNU GPL
PSOVIS	Data visualizations only	-	-	EyeLink	GPL-3.0
PyGazeAnalyzer	-	-	-	Eye tracker agnostic	GPL-3.0
ETRAN—R	-	-	-	SMI	CC BY-NC 4.0
BEACH-Gaze	✓	✓	✓	Gazepoint	GNU GPL

**Table 2:** Descriptive Gaze Measures.

Gaze Attribute	Descriptive Measure	Definition
Saccades	Count	The total number of valid saccades
	Magnitude (px)	The sum, mean, median, SD, minimum, and maximum distance between fixations
	Duration (s)	The sum, mean, median, SD, minimum, and maximum duration of the saccades
	Amplitude (°)	The sum, mean, median, SD, minimum, and maximum angular distance the eye travels during saccades
	Velocity (°/s)	The sum, mean, median, SD, minimum, and maximum speed of eye movements during saccades
	Peak velocity (°/s)	The sum, mean, median, SD, minimum, and maximum of the highest speeds of eye movements during saccades
	Relative direction (°)	The sum, mean, median, SD, minimum, and maximum angle between two saccades
	Absolute direction (°)	The sum, mean, median, SD, minimum, and maximum angle of the saccade with respect to the horizontal axis
Fixations	Count	The total number of valid fixations
	Duration (s)	The sum, mean, median, SD, minimum, and maximum duration of the fixations
	Convex hull (px^2^)	The smallest area that encompasses all fixations
AOI specific	Stationary entropy	The distribution of a person’s gaze across AOIs
	Transition entropy	The extent to which a person has transitioned between the different AOIs
	Proportion of fixations within AOI	The percentage of fixation counts that occurred in an AOI.
	Proportion of fixation duration within AOI	The percentage of fixation durations that occurred in an AOI
	AOI pair	Identifies the two AOIs where a gaze transitions from the first to the second in sequence
	Transition count	The number of transitions that occurred for an AOI pair
	Proportion of transition including self-transitions	The proportion of total transitions that occurred between AOI pairs, inclusive of self-transitions
	Proportion of transition excluding self-transitions	The proportion of total transitions that occurred between AOI pairs, exclusive of self-transitions
	Pattern of AOI	A string of characters representing a sequence of AOIs, either expanded or collapsed
	Pattern frequency	The number of times a pattern appears in a person’s sequence of AOIs visited, either expanded or collapsed
	Sequence support	The proportion of sequences in a user group where the pattern appears, relative to the total number of sequences identified for that group, either expanded or collapsed
	Mean pattern frequency	The total occurrences of the AOI pattern across all sequences in a user group, divided by the group’s total number of sequences, either expanded or collapsed
	Proportional pattern frequency	The proportion of total AOI pattern occurrences within a group relative to the group’s overall number of patterns, either expanded or collapsed
Blinks	Average blink rate (blink/m)	The average rate at which a person blinks per minute
Pupil	Left eye average pupil size (mm)	The average pupil size of the left eye
	Right eye average pupil size (mm)	The average pupil size of the right eye
	Average pupil size across both eyes (mm)	The average pupil size across both eyes
Combined	Fixation-to-saccade Ratio	The sum of fixation duration divided by the sum of saccadic duration
	Scanpath duration (s)	The duration of all fixations and saccades

**Table 3:** Classification and Regression Models Supported in BEACH-Gaze.

Type	Model
Classification	Zero Rule; Bayesian Network; Naive Bayes; Naive Bayes Multinomial Text; Naive Bayes Updateable; Naive Bayes Multinomial; Naive Bayes Multinomial Updateable; Logistic Regression; Multilayer Perceptron; Stochastic Gradient Descent; Stochastic Gradient Descent Text; Simple Logistic Regression; Sequential Minimal Optimization (for Support Vector Machines); Voted Perceptron; Instance-Based k (k-Nearest Neighbors); K* (K-Star); Locally Weighted Learning; Adaptive Boosting Method 1; Attribute Selected Classifier; Bootstrap Aggregating; Classification via Regression; Cross-Validation Parameter Selection; Filtered Classifier; Iterative Classifier Optimizer; Logistic Boosting; Multi-Class Classifier; Multi-Class Classifier Updateable; Multi-Scheme; Random Committee; Randomizable Filtered Classifier; Random Subspace; Stacking; Vote; Weighted Instances Handler Wrapper; Input Mapped Classifier; Decision Table; Repeated Incremental Pruning to Produce Error Reduction (RIPPER); One Rule; Partial Decision Trees; Decision Stump; Hoeffding Tree; C4.5 Decision Tree; Logistic Model Trees; Random Forest; Random Tree; Reduced Error Pruning Tree.
Regression	Zero Rule; Gaussian Processes; Linear Regression; Multilayer Perceptron; Simple Linear Regression; Sequential Minimal Optimization for Regression; Bootstrap Aggregating; Cross-Validation Parameter Selection; Regression By Discretization; Multiple Scheme; Random Committee; Randomizable Filtered Classifier; Random Subspace; Stacked Generalization; Voting; Weighted Instances Handler Wrapper; Decision Table; M5 Rules; M5 Prime; Reduced Error Pruning Tree; Instance-Based k-Nearest Neighbors; K* (K-Star); Locally Weighted Learning; Decision Stump; Random Forest; Additive Regression; Attribute Selected Classifier; Elastic Net; Isotonic Regression; Least Median of Squares; Iterative Absolute Error Regression.

## Data Availability

The data presented in this study are not openly available due to privacy and ethical restrictions. Requests to access the data should be directed to the lead author.

## Abbreviations

The following abbreviations are used in this manuscript:

BEACH-Gaze	Beach Environment for the Analytics of Human Gaze
AOI	area of interest
GUI	graphical user interface
DGM	descriptive gaze measure
SD	standard deviation
WEKA	Waikato Environment for Knowledge Analysis
ASD	autism spectrum disorder
BPM	beats per minute
ILS	Instrument Landing System
